# The miRNA-184 drives renal fibrosis by targeting HIF1AN in vitro and in vivo

**DOI:** 10.1007/s11255-018-2025-4

**Published:** 2018-12-10

**Authors:** Bin Chen

**Affiliations:** Kidney Department, Zhenhai People’s Hospital of Ningbo City (Ningbo No.7 Hospital), 718 Nanerxi Road, Luotuo Subdistrict, Zhenhai, Ningbo, People’s Republic of China

**Keywords:** MiRNA-184, HIF1AN, Renal fibrosis

## Abstract

Progressive renal fibrosis is the last phase of chronic kidney disease and results in renal failure. Micro-RNA has been demonstrated as important agent to drive organ fibrosis. However, the precise mechanisms are not fully understood. Here, we found miRNA-184 as a critical mediator to promote the renal fibrosis by targeting HIF1AN. In Vivo, miRNA-184 expression levels remarkably increased both in patients’ serum and in unilateral ureteral obstruction kidneys, as well as induced the expression of COL1A1 and COL3A1. Furthermore, transfection of NRK49F cells with miRNA-184 mimics down-regulated HIF1AN, transfection of NRK49F cells with miRNA-184 inhibitor up-regulated HIF1AN, while the cells transfected with miRNA-184 inhibitor exerted the opposite effect. When the cells were co-transfected with miRNA-184 mimics and HIF1AN, the expression of α-SMA, GTGF, COL1A1, and COL3A1 at mRNA level was apparently decreased when compared with miRNA-184 mimic-transfected cells, which was strengthened when transfected with miRNA-184 inhibitor. Thus, miRNA-184 is an important agent to promote the fibrosis though binding to HIF1AN, and may be a promising novel target in treatment of renal fibrosis.

## Introduction

Renal fibrosis is the final stage that manifesting the chronic kidney disease and leading to impairment of kidney function [1, 2]. A typical hallmark of tubule interstitial fibrosis is composed of myofibroblast accumulation, excessive deposition of extracellular matrix (ECM) as well as the renal tubules destruction [3, 4]. Other critical characteristics include accumulation of inflammatory leucocytes, which causes loss of renal function further [5, 6]. Fibrosis is key element of the high morbidity and mortality rates, especially related to some diseases, such as diabetic nephropathy. However, concrete therapeutic options for this target are not yet available in clinical application.

Recent studies show that miRNA can be involved in regulating numerous molecular and cellular processes, as well as its role in diseases and therapeutic potential [7–10]. Micro-RNAs are a class of short noncoding RNAs of approximate 22 nucleotides in length, and they can regulate gene expression via post-translation and induction of mRNA degradation [11]. A certain number of miRNA have been considered to be associated with fibrotic processes in some relevant diseases, including miR-29, miR-192, and miR-21 [12–15]. It’s reported that these miRNAs are induced by TGF-ß1 in renal cells [16], but another study demonstrates that TGF-ß1 deduces miR-192 expression in human tubular epithelial cells [17]. The normalization of their expression could alleviate fibrosis in vitro and in vivo model, which suggests that focusing on these miRNAs could be a method to improve renal fibrosis disease [16].

Therefore, our study aimed to investigate miRNA profiling in the kidneys, and also to identify the potential mechanism in renal fibrosis. Moreover, we carried out intervention studies to determine the role of miRNA-184 in promoting the renal fibrosis. Finally, we determined whether suppressing the H1F1 might be a target by which miRNA-184 regulates renal impairment.

## Materials and methods

### Experimental animals

A UUO kidney disease model was induced in C57 mice by left ureteral ligation in accordance with a previously described method [18]. After raising for 1 week, they were randomly divided into four group, one was sham surgery group with 10% DMSO intraperitoneal injection as control, and others were UUO models with CORM at doses of 2.5 mg/kg, 10 mg/kg, and 15 mg/kg, respectively, each group of 6 mice. Mice were killed after administration for 14 days, and the supernatant from eyeball blood was collected though centrifugation and stored at − 80º. Kidney tissues were collected and processed for evaluation of miR-184 as well as for immunohistochemistry, western blot, and real time PCR.

### Cell culture and transfection

NRK49F cells were cultured in Dulbecco’s modified Eagle’s medium, 10% fetal calf serum (FCS), 1% glutamine, 1% nonessential amino acids, and antibiotics at 37 °C, 5% CO_2_ under humidified conditions. For the first time, we used Angiotensin II (R&D Systems) to stimulate the cells to determine miRNA-184 expression levels. Moreover, NRK49F cells were respectively transfected with 30 nM miRNA-184 mimics (Ambion, Austin, TX), miRNA-184 inhibitor (Ambion, Austin, TX), negative control miRNA mimics (Ambion), or miRNA-184 inhibitor in six well plates using siPort Neo-FX (Ambion) according to the manufacturer’s instructions. After transient transfection, the cells were synchronized by culturing them in low-glucose medium without serum for 24 h. NRK49F cells were then stimulated with AngII in accordance with our protocols.

### Quantitative RT-PCR

The assay was carried out as previously described [19]. Quantitative RT (qRT)-PCR analyses of miR-184, HIF1AN mRNA, α-SMA mRNA, GTGF mRNA, COL1A1 mRNA, and COL3A1 mRNA were carried out in RNA isolated from patients’ serum or mice kidney tissue using specific TaqMan assays (Life Technologies). GAPDH was used as an internal control.

### Luciferase reporter assays

NRK49F cells were co-transfected with a construct containing the human HIF1AN 3′untranslated region (UTR) downstream of the Firefly luciferase gene, the co-reporter vector pMIR-REPORT encoding the Renilla luciferase and miR-184 mimics. After culturing for 36 h, the activity of biluciferase was analyzed after cells were lysed with the passive lysis buffer by the Dual-Luciferase Reporter Assay System (Promega, Madison, WI, USA).

### Western blot

Transfected NRK49F cells were also collected according to our protocols. Western blot analysis was carried out as previously described [20, 21], with primary antibodies against α-SMA (Sigma, US) and HIF1AN (Sigma, US). GAPDH was used as an internal control.

### Histology

Changes in renal morphology were examined in H&E and MASSON staining. The kidney tissues were fixed, fully automatic dehydrated, paraffin-embedded, and sliced into tissue sections (4 mm). All the slices were stained with hematoxylin for nuclei, and Masson’s trichrome stain (Solarbio, China) was performed according to the manufacturer’s specific instructions.

## Results

### The miRNA-184 expression levels in vivo

**Fig. 1:**
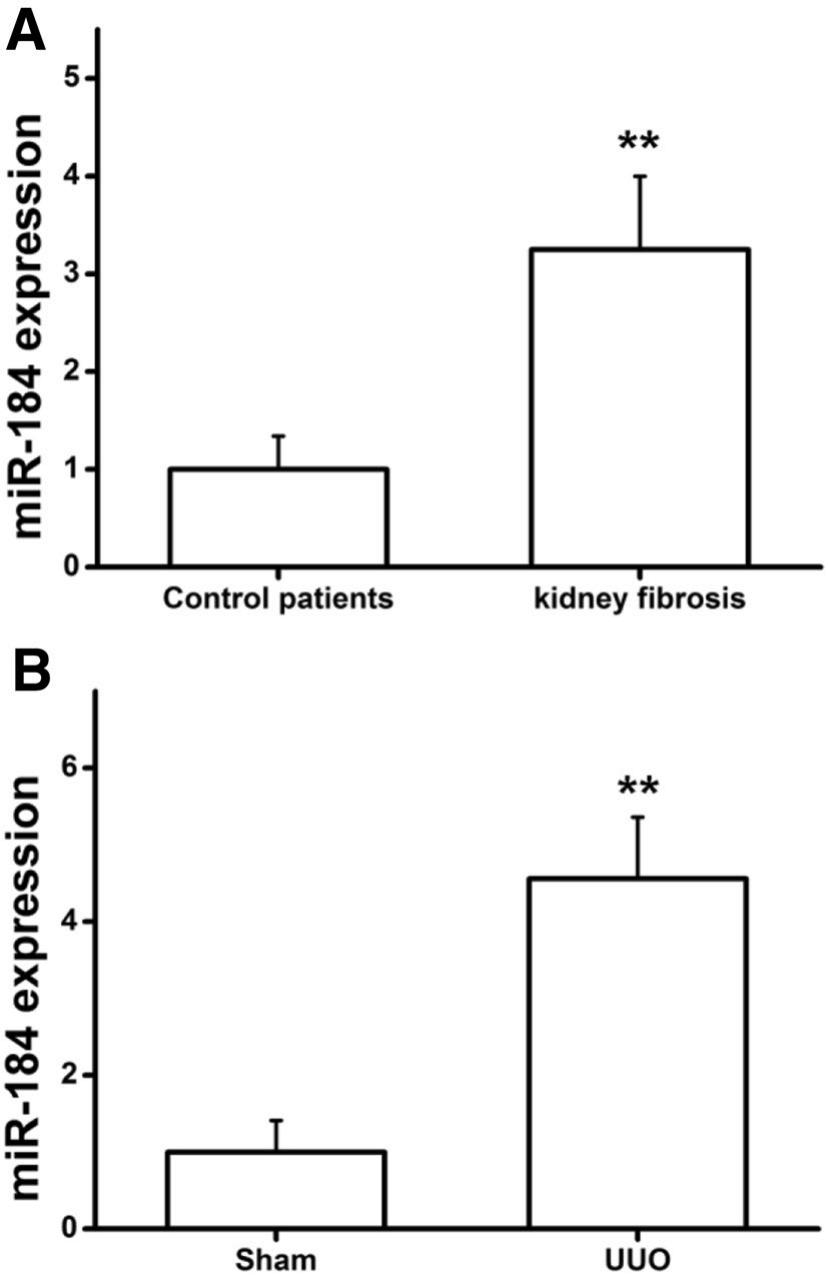
MiRNA-184 expression levels in vivo. **a** miRNA-184 expression levels in both observation group and health group were determined by qRT-PCR assays. **b** miRNA-184 expression levels in mice kidney of sham operation group and of UUO group were determined by qRT-PCR assays. **Statistically highly significant, *P* < 0.01

To determine whether miRNA-184 plays a role in the progression of kidney fibrosis, we detected the miRNA-184 expression levels in serum from both health group and observation group with kidney fibrosis, and to analyze the differences between the two groups. The results showed that miRNA-184 expression levels were significantly increased compared with the health group as shown in Fig. 1a. Meanwhile, we used the qRT-PCR to detect the levels of miRNA-184 in mice kidney of sham operation group and of unilateral ureteral obstruction (UUO) group. Of note, the miRNA-184 expression levels in UUO group were significantly increased than those in the sham operation group as shown in Fig. 1b. These data indicated that miRNA-184 may play a key role in the development of fibrotic responses in the kidney.

### MiRNA-184 induces α-SMA expression in vitro

**Fig. 2:**
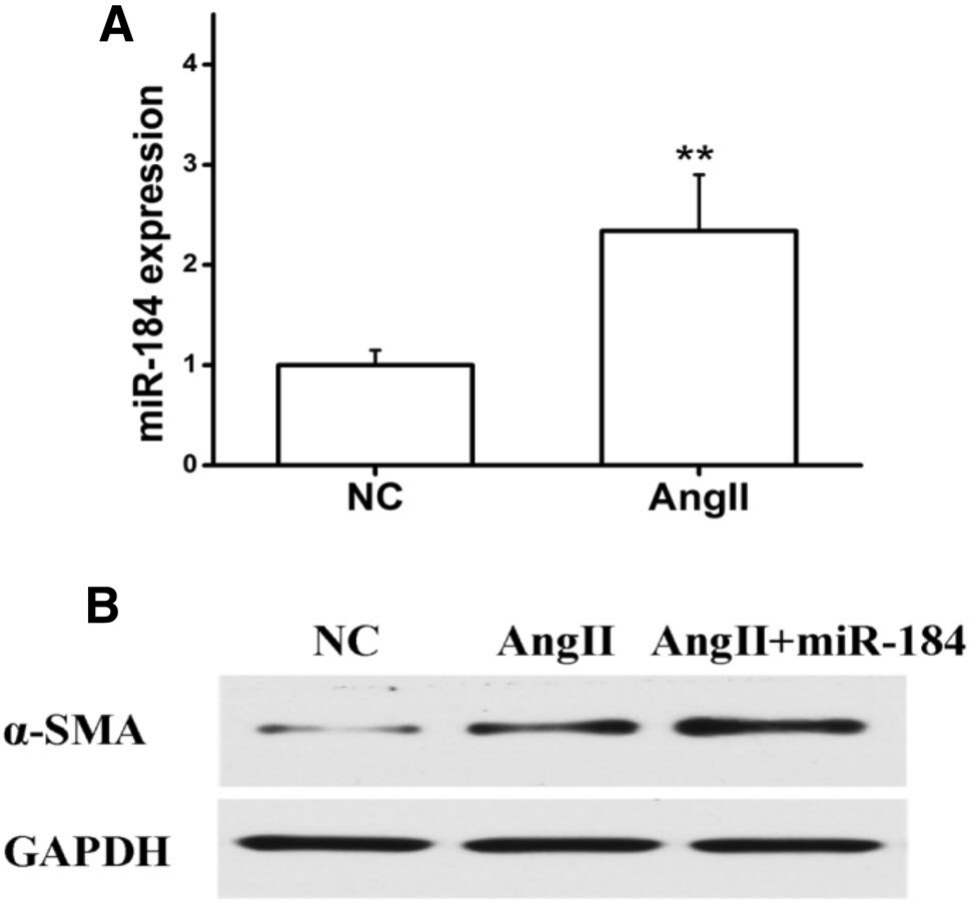
MiRNA-184 induces α-SMA expression in vitro. **a** miRNA-184 expression levels stimulated with AngII were determined by qRT-PCR assays. **b** α-SMA expression levels stimulated with AngII and AngII + miRNA-184 were determined by Western blotting analysis. **Statistically highly significant, *P* < 0.01

We used NRK49F, a rat renal fibroblasts line, for mechanistic study to causal model of miRNA-184 on renal fibrosis. Angiotensin II, albuminuria and TGF-β could contribute to tubule interstitial impairment and fibrosis in renal diseases [22, 23]. In order to investigate whether the stimuli could trigger miR-184 expression in renal fibroblasts line, we exposed NRK49F cells to AngII for 36 h. Quantity RT-PCR results showed that miRNA expression levels were apparently increased after NRK49F was stimulated with AngII, as shown in Fig. 2a. Transfection of NRK49F cells with miRNA-184 mimics and under stimulation with AngII resulted in a significant increase in α-SMA compared with negative control mimic-transfected cells. Western blotting analysis indicated that miRNA-184 could cause a sustained up-regulation of the α-SMA expression **(**Fig. 2b**)**.

### HIF1AN is a target of miRNA-184

**Fig. 3:**
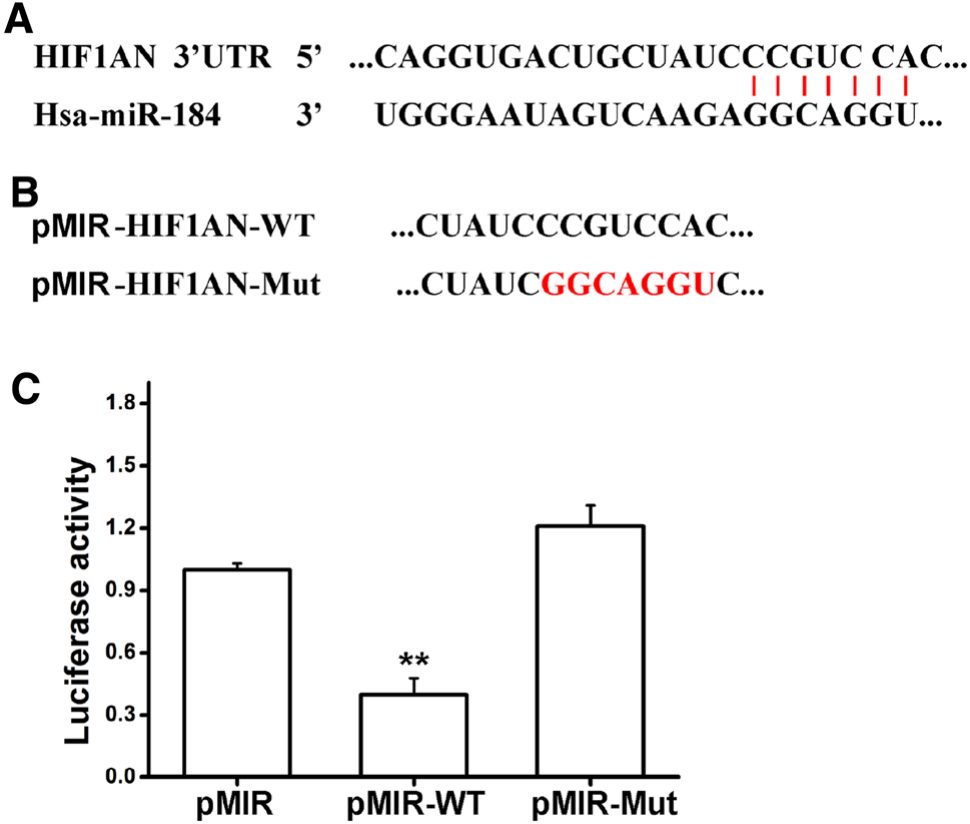
HIF1AN is a target of miRNA-184. **a** 3′-UTR of HIF1AN binding to miRNA-184 as indicated. **b** The plasmid pMIR-HIF1AN-mut was constructed by overlapping PCR assay. **c** Luciferase activity in NRK49F cells co-transfected with the reporter plasmid containing the HIF1AN 3′-UTR downstream of the Firefly luciferase gene, the co-reporter vector pMIR-REPORT encoding the Renilla luciferase and with miR-184 mimic. **Statistically highly significant, *P* < 0.01

To demonstrate the target gene of miRNA-184, miRNA target prediction algorithms miRanda and EIMMo were employed together with the micro-RNA body map web tool. We focused on the HIF1AN (Fig. 3a) because we investigated the micro-RNA target gene database and made such a prediction. In order to prove that HIF1AN is a true miRNA-184 target, we carried out luciferase reporter assay employing a plasmid pMIR-REPORT with full-length 3′-UTR of HIF1AN downstream of the luciferase gene. When the plasmid named pMIR-HIF1AN-wt was built, we performed overlapping PCR assay to construct plasmid pMIR-HIF1AN-mut (Fig. 3b**)**. To detect whether miRNA-184 could regulate the HIF1AN expression, NRF49F cells were co-transfected with miRNA-184 mimics and reporter plasmid. As shown in Fig. 3c, compared with pMIR-REPORT cells, it was showed that luciferase activity of pMIR-HIF1AN-mut was significant decreased, but that of pMIR-HIF1AN-mut had no statistical changes. Taken together, these results suggested that miRNA-184 could suppress HIF1AN expression by binding 3′-UTR of HIF1AN.

### MiRNA-184 regulates HIF1AN expression in mRNA and protein levels

**Fig. 4:**
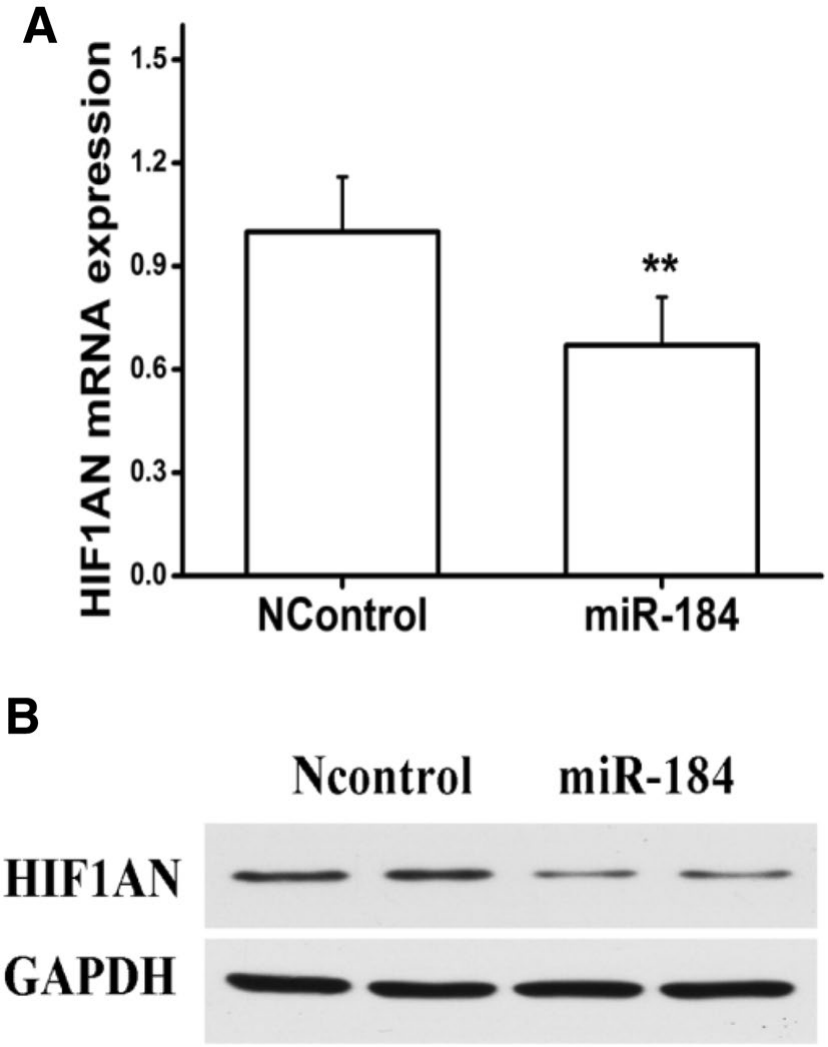
MiRNA-184 regulates HIF1AN expression in mRNA and protein levels. Co-transfection of NRF49F cells with miRNA-184 mimics and negative control miRNA mimic. **a** HIF1AN mRNA expression was suppressed by miRNA-184. **b** HIF1AN protein expression was suppressed by miRNA-184. **Statistically highly significant, *P* < 0.01

**Fig. 5:**
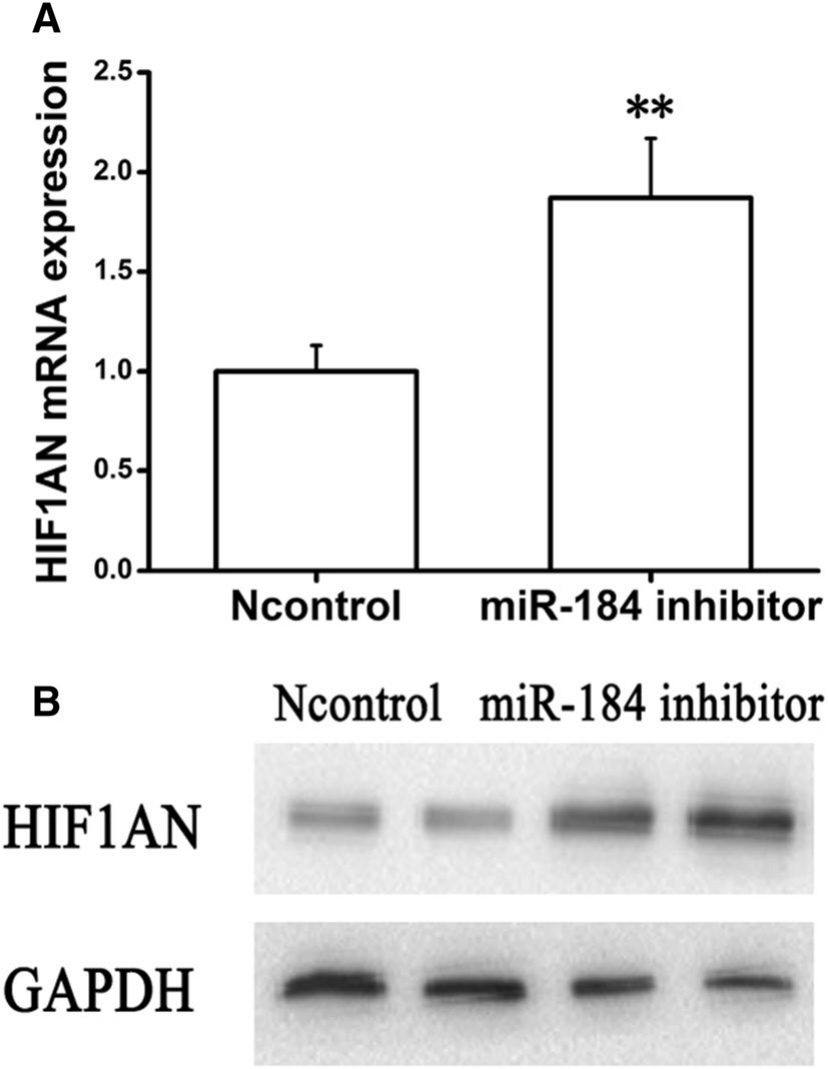
MiRNA-184 inhibitor regulates HIF1AN expression in mRNA and protein levels. Co-transfection of NRF49F cells with miRNA-184 inhibitor or negative control miRNA mimic. **a** HIF1AN mRNA expression was up-regulated by miRNA-184 inhibitor. **b** HIF1AN protein expression was up-regulated by miRNA-184 inhibitor. **Statistically highly significant, *P* < 0.01

To estimate whether miRNA-184 could regulate HIF1AN in mRNA levels, we performed qRT-PCR assay to determine the mRNA levels of HIF1AN. As shown in Fig. 4a, transfection of NRF49F cells with miRNA-184 mimics showed a significant reduction in HIF1AN expression at mRNA levels compared with negative control miRNA mimic-transfected cells. As shown in Fig. 5a, transfection of NRF49F cells with miRNA-184 inhibitor showed a significant increase in HIF1AN mRNA compared with negative control miRNA mimic-transfected cells. Meanwhile, we carried out Western blot assay to detect the expression levels of HIF1AN. As shown in Fig. 4b, consistent with HIF1AN mRNA down-regulation, HIF1AN protein expression of NRF49F cells transfected with miRNA-184 mimics resulted in a significant reduction compared with negative control miRNA mimic-transfected cells. As shown in Fig. 5b, HIF1AN protein expression of NRF49F cells transfected with miRNA-184 inhibitor resulted in a significant increase compared with negative control miRNA mimic-transfected cells. Based on the above results, we found that miRNA-184 could down-regulate HIF1AN expression in mRNA and protein levels.

### MiRNA-184 promotes the progression of renal fibrosis inhibited by HIF1AN

**Fig. 6:**
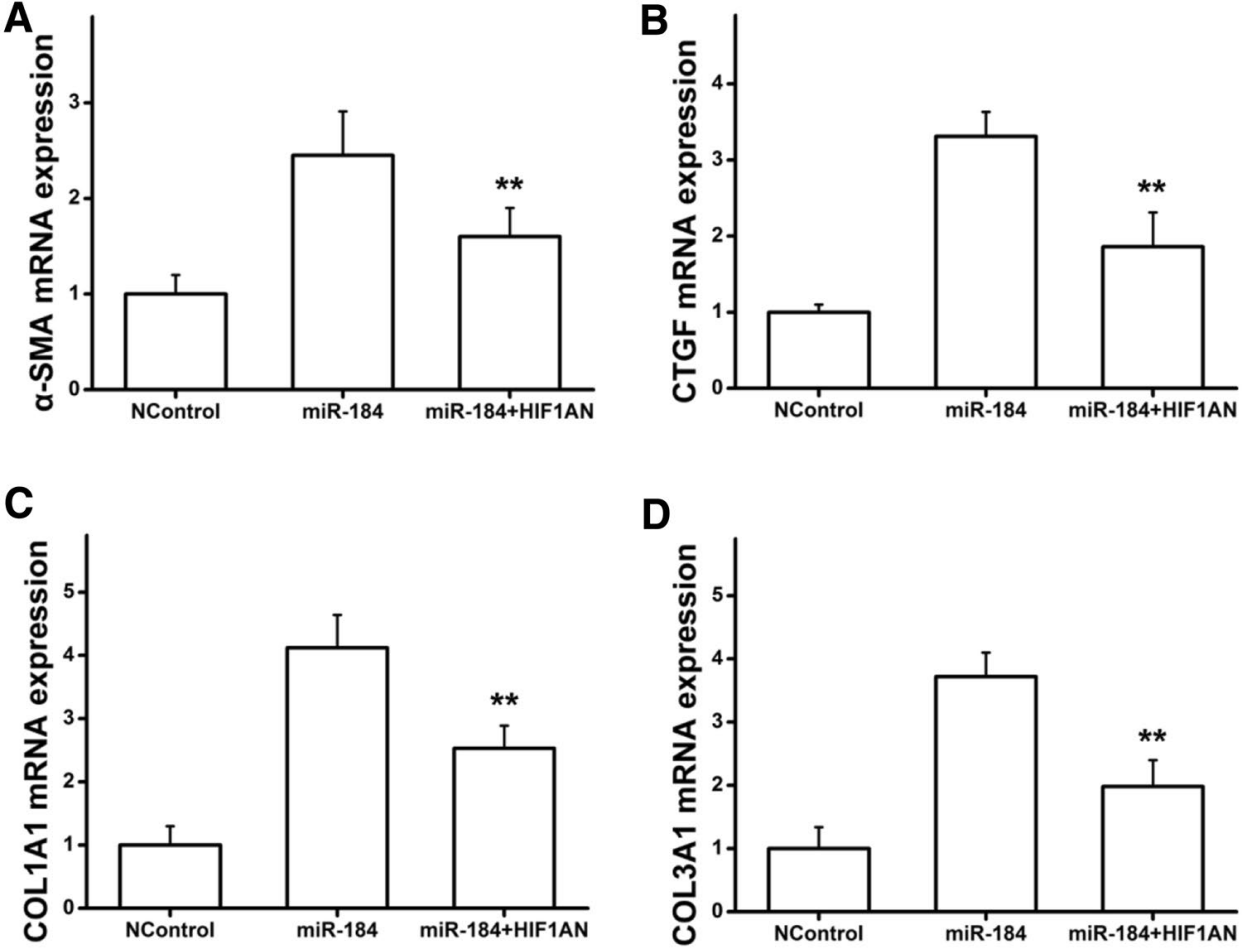
MiRNA-184 promotes the progression of renal fibrosis inhibited by HIF1AN. Co-transfection of NRF49F cells with miRNA-184 mimics, negative control miRNA mimic and miRNA-184 mimics + HIF1AN. **a** α-SMA mRNA expression. **b** GTGF mRNA expression. **c** COL1A1 mRNA expression. **d** COL3A1 mRNA expression. **Statistically highly significant, *P* < 0.01

**Fig. 7:**
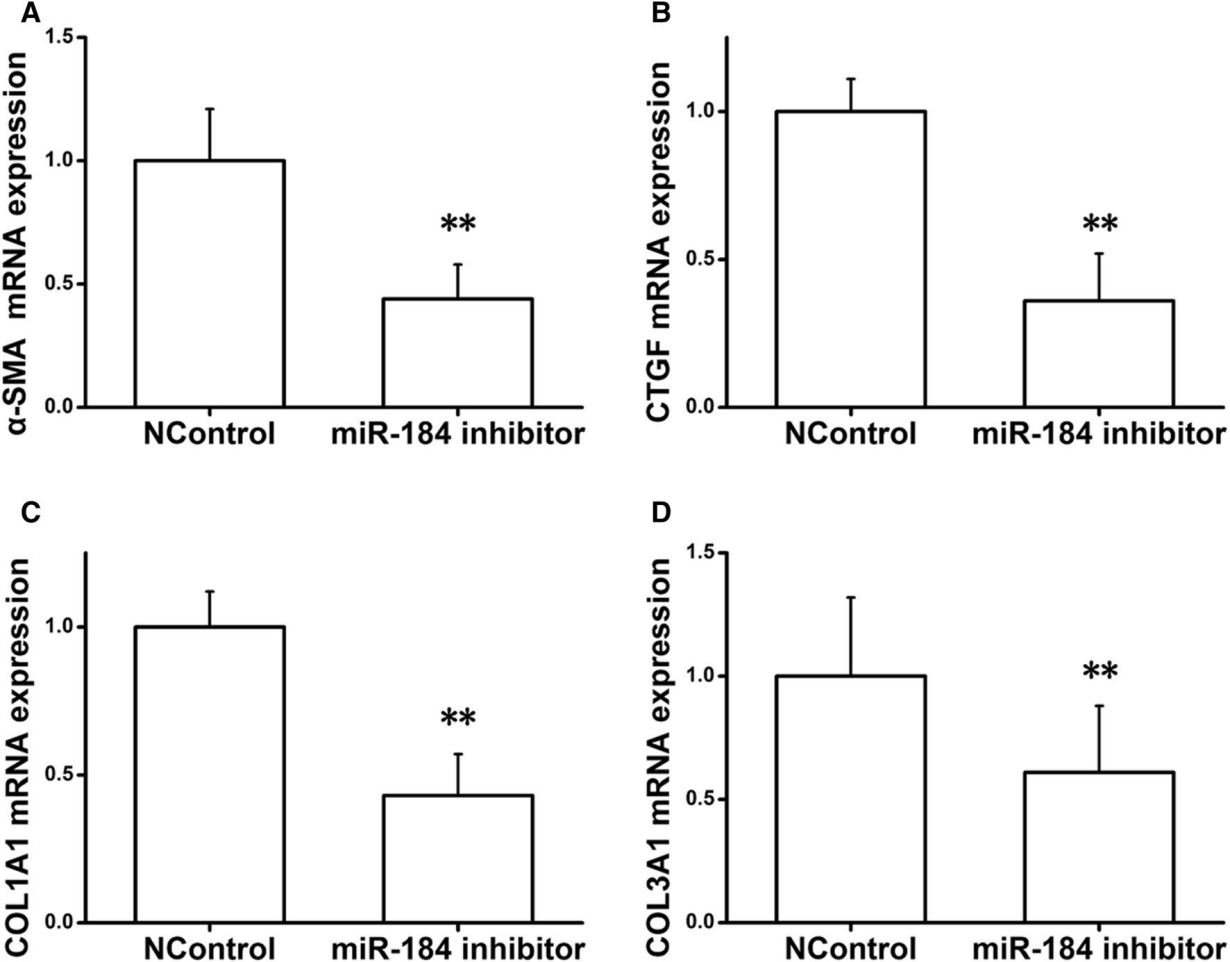
The effect miRNA-184 inhibitor at miRNA expression levels of α-SMA, GTGF, COL1A1 and COL3A1. Co-transfection of NRF49F cells with miRNA-184 inhibitor. **a** α-SMA mRNA expression. **b** GTGF mRNA expression. **c** COL1A1 mRNA expression. **d** COL3A1 mRNA expression. **Statistically highly significant, *P* < 0.01

In order to validate that miRNA-184 promoted the progression of renal fibrosis by inhibiting HIF1AN expression, qRT-PCR assay was performed to determine the expression of α-SMA, GTGF, COL1A1, and COL3A1. We identified that above indictors in cells transfected with miRNA-184 mimics were significantly increased compared with negative control miRNA mimic-transfected cells. However, α-SMA, GTGF, COL1A1, and COL3A1 mRNA expression in cells co-transfected with miRNA-184 mimics and HIF1AN were apparently decreased when compared with miRNA-184 mimics-transfected cells (Fig. 6a–d). In addition, the expression of above parameters were significant down-regulated when transfected with miRNA-184 inhibitor (Fig. 7a–d). Notably, miRNA-184 promoted the progression of renal fibrosis, which could be inhibited by HIF1AN. In a word, HIF1AN involves in the progression of renal fibrosis mediated by miRNA-184.

### Renal fibrosis morphology in UUO mice associated with miRNA-184 modulation

**Fig. 8:**
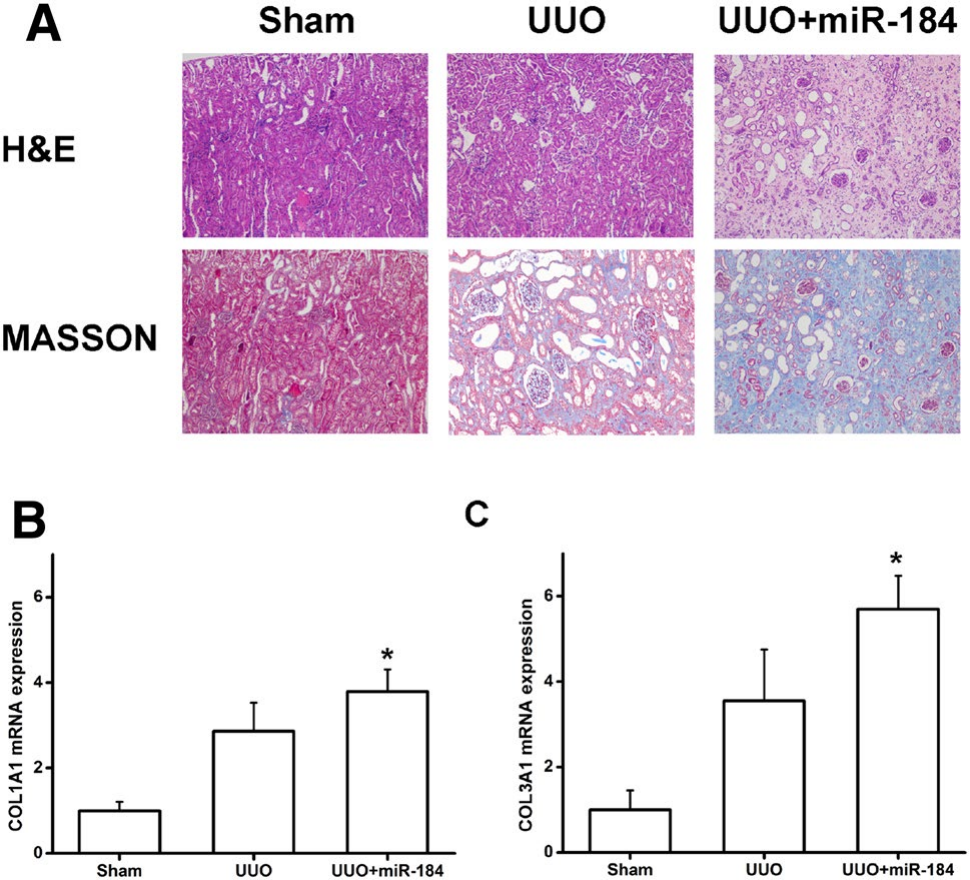
Renal fibrosis morphology in UUO mice associated with miRNA-184 modulation. UUO mice model was established and make an injection of miRNA-184 into the tail vein. **a** HE and MASSON staining of mice kidney in various groups. **b** COL1A1 mRNA expression. **c** COL3A1 mRNA expression. *Statistically significant, *P* < 0.05

Based on the in vitro data indicating the effect of miRNA-184 in the progression of renal fibrosis, we moved towards in vivo study to demonstrate the miRNA-184 regulated the consequent fibrosis. HE staining results showed that the structure of renal tubules and glomerulus were clear, the epithelial cells arranged neatly, the basement membrane of small organs was intact, and no obvious cell infiltration was observed in the interstitium in the sham-operated mice. However, in the UUO mice, there was renal glomerular dilatation, epithelial cell degeneration, and necrosis, indicating UUO kidney was successfully modeled. The injection of miRNA-184 into the tail vein resulted in more pronounced renal tubular dilatation and increased degeneration and necrosis of epithelial cells. Moreover, we analyzed the changes of renal fibrosis in mice performing Masson’s trichrome staining. The results showed that the area of renal fibrosis and extracellular matrix deposition were significantly increased in UUO mice with miRNA-184 injection compared with sham-operated mice and UUO mice (Fig. 8a). Consistent with in vitro results of COL1A1 and COL3A1 mRNA expression, we found that the expression levels of COL1A1 and COL3A1 mRNA in sham-operated mice and UUO mice were significantly decreased than those in UUO mice injected with miRNA-184 (Fig. 8b, c). The above results showed that miRNA-184 could significantly promote progression of renal fibrosis.

### Statistical analysis

Descriptive statistics were calculated and shown in figures. One-way ANOVA analysis was performed for multiple group comparisons. The Student’s *t*-test was used for comparison between two groups. *P* values less than 0.05 was considered statistically significant difference. The statistical analysis was performed using SPSS software (SPSS for windows 17.0, SPSS Inc.).

## Discussion

Renal fibrosis is the final common stage of any form of progressive kidney disease, resulting in renal impairment. For a long-term study, we have been exploring the relevant molecular mechanisms involving in the development of renal fibrosis and identified that the miR-324-3p/Prep complex and miR-433 exerted effect during the fibrotic process [24, 25]. Here, we focused on miRNA-184 and demonstrated that is a key agent during the renal fibrosis.

Little information is available pertaining to miRNA-184 expression in the kidney. Some reporters showed that renal miRNA profiling of rodents displayed upregulated miR-184 in aged kidneys, indicating that epigenetic regulation of renal aging likely occurred via inhibiting miRNA-184 targeted genes [26, 27]. Zan chi et al. [28] established a link between abnormal tubular miR-184 and tubule interstitial fibrosis in the diabetic kidneys through suppression of the target LPP3, which plays a critical role in modulating biosynthesis of lipid phosphates involved in multi-organ fibrosis and in cell signal transduction [29, 30]. In this study, we added new finding that miRNA-184 is a positive agent binding HIF1AN as a potential target during the fibrosis. HIF1AN, an asparaginyl (Asn) hydroxylase, is able to modulate the activities of important biological regulators (such as HIF1a [31], IĸB [32], Notch [33]) through hydroxylation. It has been reported that HIF-1α exerted an effect in hypoxia- and chronic kidney disease-induced fibrosis, while genetic knock-down of HIF-1α in renal epithelial cells led to a significant reduction in collagen deposition, including COL1A1 and COL3A1 [34]. Mahon et al. revealed that HIF1AN considered as an important inhibitor can interact with Hypoxia-inducible factor (HIF)-1α to negatively regulate the HIF-1α transcriptional activity [35]. Therefore, HIF1AN can be regarded as a critical agent implicated in the process of renal fibrosis.

It’s reported that Micro-RNAs suppress their target gene though binding their 3′ UTR to induce protein translational inhibition or mRNA degradation [31]. In order to identify whether miRNA-184 could target HIF1AN gene, we detected the expression levels of HIF1AN in rat kidney fibroblasts overexpressing miRNA-184 in the miRNA and protein levels. Notably, both mRNA and protein levels of HIF1AN were apparently inhibited by miRNA-184 when compared with the control (Fig. 4a, b). While both mRNA and protein levels of HIF1AN were apparently promoted by miRNA-184 inhibitor when compared with the control (Fig. 5a, b). To further determine whether miRNA-184 modulates HIF1AN by binding its 3′ UTR, we generated luciferase reporter linked to full-length 3′-UTR of HIF1AN and NRF49F cells were co-transfected with miRNA-184 mimics and reporter plasmid. Compared with pMIR-REPORT cells, it was showed that luciferase activity of pMIR-HIF1AN-wt was significantly decreased, which indicated that miRNA-184 suppressed its target gene HIF1AN expression by specifically binding its 3′-UTR. Above findings of target gene of miRNA-184 were consistent with the theory that the target sites of micro-RNA could be enriched in multiple components of the same signaling pathway [36, 37].

Angotens in II is a critical mediator of proteinuria and fibrosis, which failed to induce miRNA-184 expression in rat kidney cells. It’s able to express angiotensin II type 1 receptors [38], suggesting that angiotensin II is lack of a direct effect on miRNA-184. In contrast, one interesting finding of our study is that Angiotensin II is an effective trigger for miRNA-184 expression in rat kidney fibroblasts (Fig. 2a). In addition, Western blotting analysis revealed that miRNA-184 could cause a sustained up-regulation of the α-SMA, CTGF, COL1A1, and COL3A1 expression (Figs. 2b, 6a, b). And miRNA-184 inhibitor could cause a sustained down-regulation of the α-SMA, CTGF, COL1A1 and COL3A1 expression (Fig. 7a, b). The fibrotic markers, such as collagens [39–41], could be induced involved in the signaling conduction pathway to promote the fibrosis and could be control involved in the signaling conduction pathway to suppress the fibrosis. Previous studies [42–44] described that TGFβ1 enhanced α-SMA, COL1A1, COL3A1, and CTGF levels to drive renal fibrosis mediated by the miR-433. In cardiac fibrosis, elevated COL3A1 has been described in early myocardial remodeling, while COL1A1 accumulation is observed at a later stage [45]. Importantly, in UUO model, we confirmed that the expression levels of COL1A1 and COL3A1 mRNA in mice injected with miRNA-184 were significantly elevated (Fig. 8b), indicating miRNA-184 may play a critically pathological role in fibrosis. In addition, we had a new finding that HIF1AN, a target of miRNA-184 during renal fibrosis, exerts its ability to regulate miRNA-184-induced the expression of fibrotic markers to facilitate fibrosis.

In conclusion, the present study provides the novel finding that miRNA-184 plays a role in renal fibrosis by targeting HIF1AN. Combination of in vitro and in vivo data showed the opportunity that targeting miRNA-184 in association with angiotensin II lowing medicine to have potential effect in renal injury disease. However, whether miRNA-184 is a rooted factor or it has an additional role in the development of renal fibrosis needs to be further studied.
